# Settlement Dynamics and Hierarchy from Agent Decision-Making: a Method Derived from Entropy Maximization

**DOI:** 10.1007/s10816-014-9219-6

**Published:** 2014-09-16

**Authors:** Mark Altaweel

**Affiliations:** Institute of Archaeology, University College London, 31-34 Gordon Square, London, WC1H 0PY UK

**Keywords:** Spatial interaction, Entropy maximization, Agent based, Modeling, Urbanism, Complexity

## Abstract

This paper presents an agent-based complex system simulation of settlement structure change using methods derived from entropy maximization modeling. The approach is applied to model the movement of people and goods in urban settings to study how settlement size hierarchy develops. While entropy maximization is well known for assessing settlement structure change over different spatiotemporal settings, approaches have rarely attempted to develop and apply this methodology to understand how individual and household decisions may affect settlement size distributions. A new method developed in this paper allows individual decision-makers to chose where to settle based on social-environmental factors, evaluate settlements based on geography and relative benefits, while retaining concepts derived from entropy maximization with settlement size affected by movement ability and site attractiveness feedbacks. To demonstrate the applicability of the theoretical and methodological approach, case study settlement patterns from the Middle Bronze (MBA) and Iron Ages (IA) in the Iraqi North Jazirah Survey (NJS) are used. Results indicate clear differences in settlement factors and household choices in simulations that lead to settlement size hierarchies comparable to the two evaluated periods. Conflict and socio-political cohesion, both their presence and absence, are suggested to have major roles in affecting the observed settlement hierarchy. More broadly, the model is made applicable for different empirically based settings, while being generalized to incorporate data uncertainty, making the model useful for understanding urbanism from top-down and bottom-up perspectives.

## Introduction

Spatial modeling of settlement rank-size hierarchies has once again become a major topic of discussion in archaeology (e.g., see Bevan and Wilson 2013; Crema 2013; Davies et al. 2014), with equation- and agent-based models (ABMs) being the most common types of approaches. This paper proposes to combine methodological contributions from ABMs and entropy maximization as a way to create a simple and a transferable model that can potentially address a variety of empirical cases derived from archaeological survey. While simulation models have enabled the actualization of processes that underline key theoretical assumptions about urbanization and settlement dynamics, relatively few case studies have integrated comprehensive and relatively intensive archaeological survey that can inform us how well model and theoretical design fit observations from the field. Such models should provide a theoretical framework to evaluate case studies and enable a quantitative-based comparison between periods to allow one to determine what underlying reasons could lead to observed rank-size hierarchies.

Various publications have applied forms of spatial interaction in assessing settlement hierarchy or site interactions (Evans 1982; Knappett et al. 2008). Spatial entropy maximizing models (Harris and Wilson 1978; Wilson 2012) have been developed to address how settlement interaction affects urban expansion or contraction. While these models have been largely applied to modern and economic settings, recent work has also applied them to past settlement systems (Bevan and Wilson 2013; Davies et al. 2014). The advantage of these methods is that they are general and accommodate a variety of case studies, including archaeological survey data at different spatial scales, and do not have complex data requirements, making them useful for cases where uncertainty prevents the understanding of specific processes that lead to observed settlement patterns. In summary, such entropy models allow the incorporation of spatial factors and feedback effects of geography, transport, and site attractiveness over a given time that enable settlement patterns to develop across a study region. Nevertheless, classical entropy maximization models do not employ individual or agent decision-making, a key factor if we are to know how theoretical complexity and complex systems from basic social units affect urban development (Adams 2001; Bentley and Maschner 2003).

This paper explores the integration of individual or agent-based methods with entropy maximization methods in understanding settlement change and settlement size hierarchy within a given region whereby households are utilized as agents. The goal of this paper is to present a simulation model that explores how the spatial setting and factors that affect individual choice result in settlement transformations and rank-size hierarchies observed in the archaeological record, while also accounting for site-specific and other regional factors that could affect settlement dynamics. Initially, background information focused on the case study is given. Then the applied methodology is introduced and discussed. Several scenarios demonstrating the model’s applicability are conducted in order to demonstrate how the model addresses the goal presented. The scenarios focus on how well model results fit the settlement size distribution, rank-size hierarchy, and account for uncertainty in settlement occupation while addressing these scenarios. Results from these scenarios are discussed, particularly how they provide insight for the research goal. The conclusion discusses broader benefits and future applications of the advanced method.

## Background

### Case Study

**Fig. 1:**
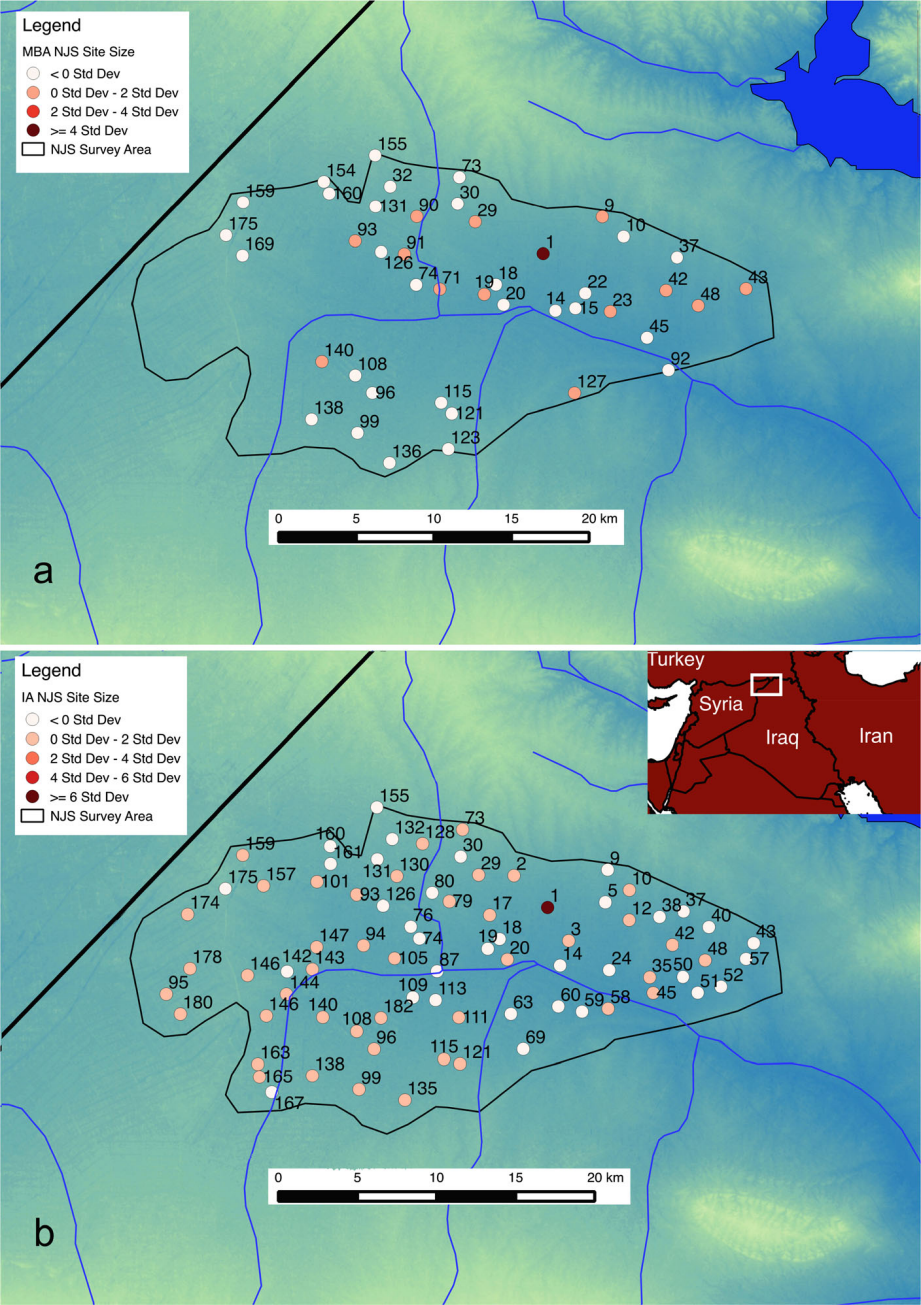
Settlements, shown by site numbers, and their sizes (ha) shown using standard deviation for the MBA (**a**) and IA (**b**) in the NJS

The North Jazirah Survey (NJS; Fig. 1; Wilkinson and Tucker 1995) provides the test case in which the applied model will be demonstrated. Because this region (~530 km^2^) has been well surveyed and a large portion of sites from various periods recovered, it serves as a useful test case. Furthermore, the area provides very different types of settlement patterns in periods studied, which could then be explored further to see what factors could have contributed to these observations.

**Fig. 2:**
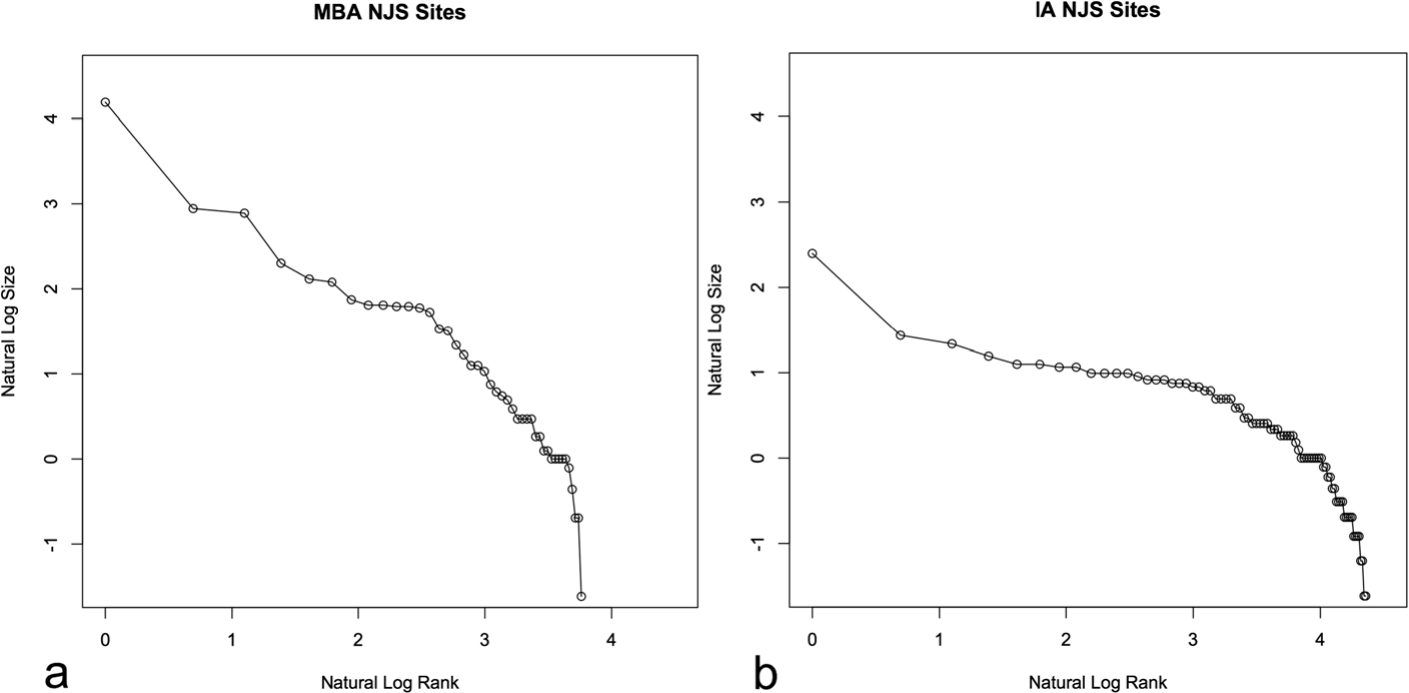
Natural log settlement rank-size distributions for the NJS sites in the MBA (**a**) and IA (**b**)

The first set of sites, 43 sites with a total area of nearly 226 ha and dominated by the site of Tell al-Hawa (≈29 % of the total area; site 1 in Fig. 1), derive from the Middle Bronze Age (MBA; 2000–1600 bc). At this time, societies across northern Mesopotamia began to develop large urban spaces and smaller settlements more intensively, while politically there was fragmentation with small states across northern Mesopotamia for much of the period (Guichard 2009). Many sites are likely to have been settled for most of this period, with excavations having indicated long-term occupation (Wilkinson and Tucker 1995; Altaweel 2006, 2007). The settlement rank-size distribution for the MBA can be given in a natural log graph (Fig. 2a), with Table 1 providing an indication of the top 10 sites for each period.

**Table 1:** Top 10 sites in size (ha) for the two periods

MBA site number	Size (ha)	IA site number	Size (ha)
1	66	1	11
93	19	138	4.2
43	18	99	3.8
127	10	108	3.3
29	8.3	3 and 140	3.0
140	8	42 and 94	2.9
9	6.5	20, 29, 48, and 174	2.7
48 and 90	6.1	163	2.6
19 and 91	6.0	58, 93, and 178	2.5
42	5.9	2, 73, and 157	2.4

While the MBA represents a period of political fragmentation whereby there was a range of major and minor settlements, the Iron Age (IA; 1200–600 bc) was a time of intensive and evenly dispersed small settlements across much of northern Mesopotamia (Wilkinson et al. 2005). In contrast to the MBA, a large territorial empire in the form of the Neo-Assyrian state characterized most of the IA in the region, which developed a highly structured provincial system of control (Radner 2006). Overall, there are 78 sites in the NJS during the IA with a total settled area of nearly 128 ha (Fig. 2b).

Measuring to see how well these distributions compare with Zipf’s law for rank-size distributions (Zipf 1949), using the Drennan and Peterson (2004) measure of deviation from Zipf’s ideal distribution, results in ≈0.58 for the MBA and ≈0.69 for the IA. This shows that the MBA sites more closely conform to the expected log-normal line following Zipf’s law for the surveyed area; however, in both cases these distributions can be characterized as convex (Johnson 1980; Savage 1997; Drennan and Peterson 2004). Statistical tests to see how different the settlement size distributions are for the two periods, using a Kolmogrov-Smirnov test and Wilcoxon ranked sum test, indicate significant differences (*p* value <0.01), even when equal subsamples are used (*p* value <0.05) to account for different overall sample sizes. In essence, the patterns for the two periods have clear quantitative and qualitative differences that demonstrate they are useful to contrast and explore what underlying processes could have caused the observed patterns to emerge.

### Theory and Empirical Constraints

Over the last two decades, ABMs applying complex system perspectives have been increasingly applied to assess both settlement patterns in case studies (e.g., Kohler et al. 2008; Kohler and Varien 2010) and abstract cases (e.g., Crema 2013). These methods generally employ individuals or households that make decisions (Epstein and Axtell 1996; Bonabeau 2002), have social connections and interact, and are affected by environmental and/or social factors. In the social sciences, ABMs are increasingly used for a wide range of social theoretical perspectives, whereby different approaches to social agency with agents ranging from rational to highly emotional actors and top-down and bottom-up social processes incorporated in research methods (Epstein 2014; Christiansen and Altaweel 2006).

To create a useful model of the past, key factors need to be considered, such as the fact that early preindustrial urban societies had relatively low rates of natural population growth (McNeil 2000). On the other hand, attractive factors, such as trade, environmental advantages, geography, economic incentive, or ideology likely lead to more rapidly increasingly populations for settlements through immigration (Desrochers 2001; Persson 2010). Furthermore, as often observed in northern Mesopotamia, settlement systems diminish and remerge over long-term cycles, with larger political entities and households connected through kinship and decision-making that affects settlement population and development (Wilkinson et al. 2007; Ur 2010).

Flexible theory explained via modeling and simulation methods need to explain how observed phenomena developed within and across a variety of periods, being able to address how observed settlement patterns are possible. To this effect, researchers have begun investigating the applicability of entropy maximizing methods that search for settlement size hierarchy and account for case study considerations, while also retaining a more abstract approach that makes the method more easily transferable to other cases (Bevan and Wilson 2013; Davies et al. 2014). Entropy maximization models have been among the most widely used urban economic or population growth models. Such models are useful at multiple scales, where neighborhoods or larger regional settlement patterns can be investigated (Wilson 1970; Harris and Wilson 1978). At a more general level, such models apply a system-theoretical approach (Casti 1985) in looking at site growth or decline. Variant forms of Lotka-Volterra equations have been applied in what are called Boltzmann-Lotka-Volterra (BLV) equations (Wilson 2008). The intent of the method is that it allows one to estimate or investigate likely areas of population growth or decline often under conditions of uncertainty. Factors of distance, economic or social relevance, including feedbacks to settlement growth, and movement capability become the generalized variables applied, with these variables encapsulating many concepts.

Nevertheless, classical entropy maximization does not look at how individuals or agents can apply decisions that may lead to settlement hierarchies observed. The intent of the new method applied here is to explore if the generalized entropy maximizing approach can incorporate some of the benefits highlighted by ABMs so that individual decision-making can be studied, rather than only using a system-level perspective, in order to address observed spatial patterns over time and still retain a method that is generalized to accommodate both uncertainty and wider applicability. Such attempts have been recently proposed and applied by Dearden and Wilson (2012), including similar methods by Birkin and Heppenstall (2011); however, to date no comparable applications have been developed to accommodate archaeological cases.

## Modeling Method

In the method applied, agents are assumed to be households of varying sizes (e.g., single-person, extended, and multi-family households). Key variables that define the model are given here, including those that are static, calculated, and given as user input. These are defined as:

- *S*_*ij*_ = calculated volume of flow (i.e., movement of people and goods) between agent *i* and settlement *j*
- *Z*_*ij*_ = calculated social-environmental attractiveness of settlement *j* to agent *i*; this represents all factors (e.g., presence of important temples, other kinship groups, etc.) that make a site attractive to settle at a given time
- *c =* operational costs, which includes bringing goods and food (e.g., via land transport) to a settlement to enable its continuity
- *d*_*ij*_ = calculated distance between two sites (*i* and *j*) based on the natural log of the cost surface
- *b*_i_ = weight for the endogenous or exogenous social benefits (e.g., trade with kinsmen), or benefit multiplier, an agent *i* has with those of similar social, cultural, or kinship backgrounds, enabling agents to be attracted to other like agents
- *t*_*j*_ *=* multiplier for endogenous or exogenous benefits (e.g., a distant state providing more goods to a settlement because of an important temple) provided for settlement *j*
- *m*_*i*_ = the probability that an agent *i* will move based on negative or relatively low flow (*S*_*ij*_)
- *α*_*j*_ = return of attractiveness for site *j* (i.e., this scales attractiveness of sites) based on social-environmental benefits (*Z*_*ij*_)
- *β* = measure of difficulty for movement (e.g., conflict limiting movement or settlement policy promoting easy movement); low to high *β* indicates decreasing to increasing impedance in movement between sites, respectively
- *u*_*j*_ = population of settlement *j*

Of these variables, five of these are user-defined inputs that are tested in scenarios, which are *α, β, m*, *c*, and *b*. The variable *u* is generally left static as the proxy used mostly as the output to compare to settlement size from surveys. In addition, *t* is generally left static (i.e., as 1.0 and all settlements are assumed to have equal benefits) and used only in scenarios where specific settlements have advantages or disadvantages that can affect agent choice. In all scenarios, distance (*d*) is determined using a cost surface analysis as defined by Fontenari et al. (2005), which accounts for elevation. This variable is calculated using ASTER (2014) terrain elevation data and measures relatively which sites are more or less costly to travel to from a given site. The variable *b* is an agent factor that could have many values, and a normal random number generator using a standard deviation to create greater variability allows for varied agent types and benefits; however, a single value is used for scenarios as this allows for an averaged value to be tested. The other variables are calculated within the simulation and discussed below.

**Fig. 3:**
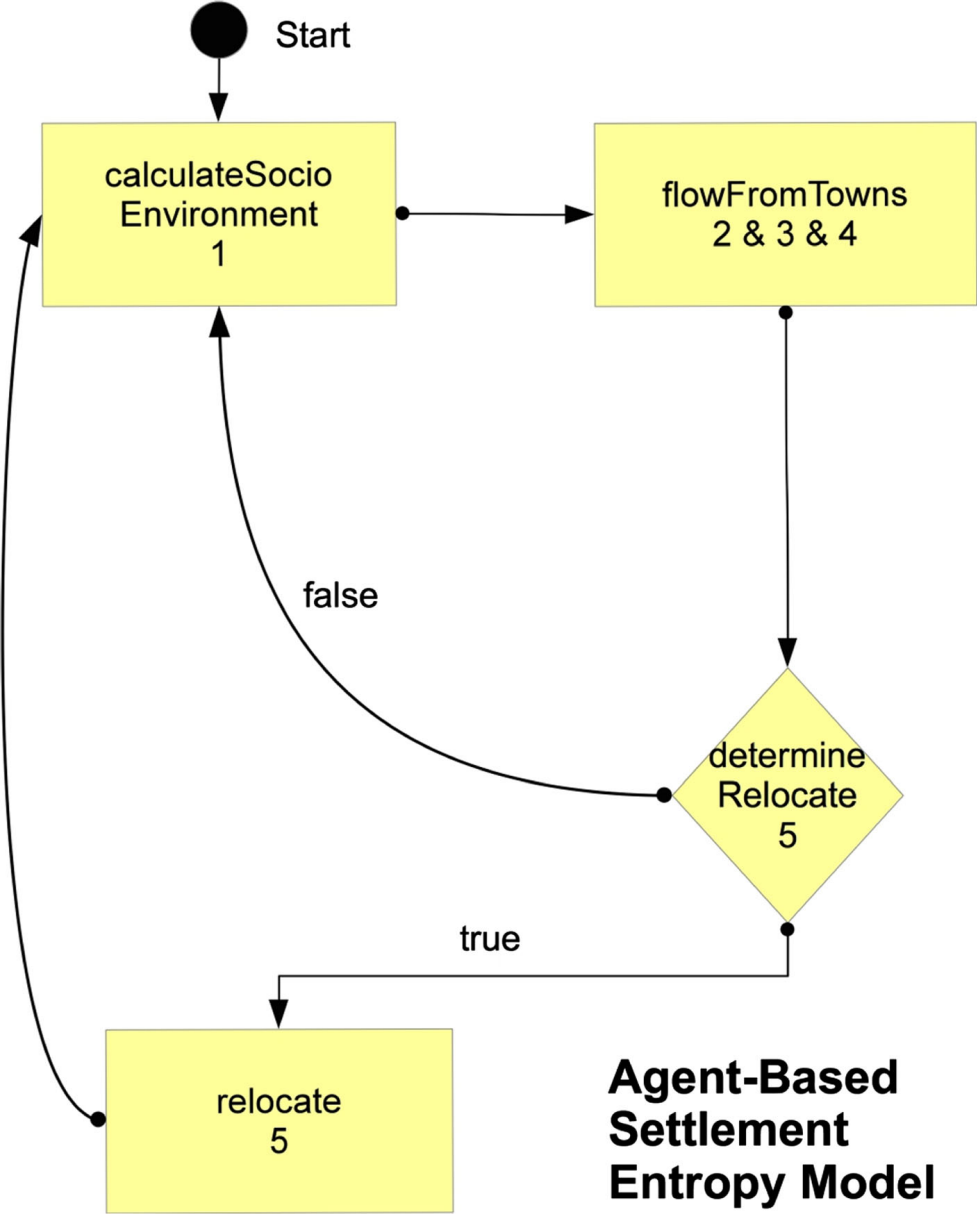
Model flowchart of operations

For the following scenarios, model operations are given below in notation and described qualitatively, provided as a downloadable code (see Electronic Supplementary Data), and demonstrated in a model flowchart (Fig. 3). The download also has additional explanations regarding how to use the model in Repast Simphony 2.1 (2014), which was used to execute the simulation, and the scenario data are also provided. The notation numbers used here are indicated in the model flowchart, while the flowchart also indicates the names of the model methods (i.e., names used in code provided) that apply the algorithms below. To begin, after the model has been initialized, the first step in the model is called calculateSocioEnvironment:

1 $$ \begin{array}{l}\left({n}_{ij}<\right)\to {Z}_{ij}={u}_j{t}_j\hfill \\ {}\left({n}_{ij}>=1\right)\to {Z}_{ij}=\left({u}_j+{n}_{ij}\right){t}_j{b}_{ij}\hfill \end{array} $$

with *Z*_*ij*_ representing attractiveness of location *j* for agent *i* based on the population (*u*) of settlement *j*, benefits of *j* (*t*), and, if there are other agents from the same social group (i.e., (*n*_*ij*_ ≥ 1)), benefits ( *b* ) these type *i* agents provide to a settlement *j*. This step is indicated as (1) in Fig. 3. This step calculates settlement attractiveness, or the reason why people want to settle in a given site, for agents based on endogenous and exogenous links the settlement has as well as those brought by agents with similar status, cultural, or kin backgrounds, with *b* controlling the relevance of this factor. This essentially allows settlements to be viewed for their sociocultural or environmental benefits, with the specifics of these purposely ignored so as to not make the model too rigid regarding a given case. The next method in the model, flowFromTowns, calls (2–4) below. Starting after the first time step, the following is calculated:

2 $$ {S}_{ij}={Z}_{ij}^{aj}{e}^{-\beta \ln \left({d}_{ij}\right)} $$

with *S*_*ij*_ representing proportion of flow, that is movement of goods and people benefiting agent *i* from settlement *j*, using *Z*_*ij*_ to the power of *α* with *e* measuring the effects of cost surface *d*, taking its natural log, and applying *β* to regulate how easy it is to move. Alpha, in essence, scales the effects of *Z*, while *β* regulates the effects of distance on movement flow. What the step does is enable distance, site attractiveness, and *β* to affect flow or goods an agent can obtain from settlements. The flow value also has a cost based on distance, which is determined below:

3 $$ {S}_{ij}^{\prime }={S}_{ij}-\left(c* \ln \left({d}_{ij}\right)\right) $$

This calculation, in effect, can limit site flow benefits to an agent based on distance and other costs. While *β* relates to the ease of movement, cost is intended to reflect production or unit costs for items or people relative to moving in a given landscape. Cost reflects ideas such as land-based transport (e.g., donkeys carrying grain to and from settlements) that have a given energy or production cost affecting flow. The aggregate of (3) for all settlements affecting agent *i* creates a net flow for *i*:

4 $$ \begin{array}{l}{D}_i={\displaystyle \sum {S}_{ij}^{\prime }}\hfill \\ {}{\mathrm{SD}}_j={\displaystyle \sum {D}_i}\hfill \end{array} $$

with (*D*_i_) being net flow for an agent and aggregate flow of all agents in a given settlement is *SD*. This provides a measure to evaluate total goods and flow an agent is getting and what the total flow is for a given settlement based on all agents in that settlement.

The next step involves the key agent-based decision-making focused on in the model, with an agent determining to find a new settlement if needed based on negative or low flow relative to other agents. This conditional, or decision made by the agent, is applied in determineRelocate in the model with the relocate method being applied if the conditional is true; if it is false the simulation returns to (1) here. For the two model methods, including the conditional, they are stated as:

5 $$ \begin{array}{l}\left(\left({D}_i<0\vee {D}_i<\overline{D}\right)\wedge m>R\right)\Rightarrow \hfill \\ {}\kern9em {\mathrm{np}}_j=1+\left({n}_{ij}/{u}_j\right)\hfill \\ {}\kern9em \left({\mathrm{SD}}_j>0\right)\to {g}_j={d}_{ij}/\left({\mathrm{SD}}_j*{\mathrm{np}}_j*{b}_{ij}\right)\hfill \\ {}\kern9em \left({\mathrm{SD}}_j<0\right)\to {g}_j=\left|{\mathrm{SD}}_j*{d}_{ij}\right|/\left({\mathrm{np}}_j*{b}_{ij}\right)\hfill \\ {}\kern9em {s}_j=\mathrm{MIN}\left({g}_j\in g\right)\hfill \end{array} $$

which determines, in the first part of this method, if negative flow or flow less than the mean flow for all agents and a probability (*R*), based on a uniform pseudorandom number generator, being less than *m* to another settlement for agent *i* results in the agent making a choice to resettle. This, essentially, allows agents to move if they are not benefiting from their current settlement or they may see their economic/social state is less desirable compared with others. The *m* factor regulates how important this is to the model. In the next part of this method, the choice of which specific settlement to move to, if the decision to move has been made, is based on the number of people (*np*) at settlement *j* that belong to the same social/kin group (*n*) as *i* relative to the settlement’s population (*u*). Then another step in this method is calculated based on if total settlement flow (*SD*) for *j* is positive or not and what the relative agent benefits (*b*) are. This calculation determines estimated benefits (*g*) for an agent based on distance and presence of social groups (*np*), including the weight of benefits  an agent has (*b*). In other words, towns, and thus other individuals in these towns, that have a higher benefit and social connection to an agent are preferred, but this could be mitigated by distance or lack of interest (i.e., low *b*) in moving to locations with similar social/kin groups. In the final step, the smallest *g* value, which in this case implies the settlement (*s*) with the greatest benefit, is selected. While using the minimal value of *g* (i.e., settlement of greatest benefit) may seem too deterministic, the variable *t*, as will be demonstrated, can allow greater variability in results.

In effect, this last step allows agents not happy with their state in their given location to migrate. If they leave, they decide where to go based on kinship/social connectivity, distance, and social-ecological factors affecting settlements’ total flow (i.e., benefits to a settlement), with these factors’ influence affected by the five user-defined inputs discussed earlier. The last and earlier methods, in fact, are all regulated by the input parameters that the simulation will test, allowing for very different circumstances to be studied for their influence on simulation results. After this step, the model returns to (1) until the end of the simulation.

## Results

The following scenarios address the primary goal of the paper that demonstrates the model’s capabilities. For scenarios, a 26-node cluster is utilized in the outputs discussed, with Repast Simphony 2.1 (2014) used for runs and R (2014) and Java Apache Commons Mathematics Library (2014) applied for statistical analysis.

### Scenario 1: Size Hierarchy Matching

The first modeling case investigates how well the ABM method can match known settlement size hierarchies from the MBA and IA, providing a general validation of the model. Ranges of input values are given in Table 2; these inputs are utilized in a parameter sweep (see North and Macal 2007) that represent qualitatively greater and lesser influences on the model, allowing one to evaluate the importance of given variables. Population (*u*) for all sites is initially set to 200; this means there are a total of 8,600 and 15,600 households for the MBA and IA subcenarios, respectively. No assumption is being made about the actual population or household sizes in the past, as the value 200 simply reflects an internal way for the model to measure relatively which sites become larger than others once people begin to migrate in the simulations. Other population values, in fact, could have been chosen, with 200 being useful to calculate population size ratios for all settlements used in outputs. What this means is that the population is used as a proxy rather than an absolute number that is then compared with settlement size (ha) as estimated from survey. In other words, the portion of the total population on a site can be directly compared with the portion of hectares out of the survey total, making the simulation and survey results comparable. All simulations are executed for 100 time ticks and up to 10 parameter runs for parameter settings, which allow results to stabilize and utilize different random seeds to account for stochasticity. Results are averaged with nearly 300,000 parameter combinations used in the subscenarios. To measure how well simulated results match empirical data, regression analysis is applied for each parameter combination.

**Table 2:** Parameters and their value ranges tested in scenario 1

Alpha (*α*)	Beta (*β*)	Cost (*c*)	Movement probability (*m*)	Benefit factor (*b*)	Initial site population (*u*)	Simulation time
0.2–10.2	0.2–10.2	0–1.0	0–1.0	1.0–11.0	200	100

**Fig. 4:**
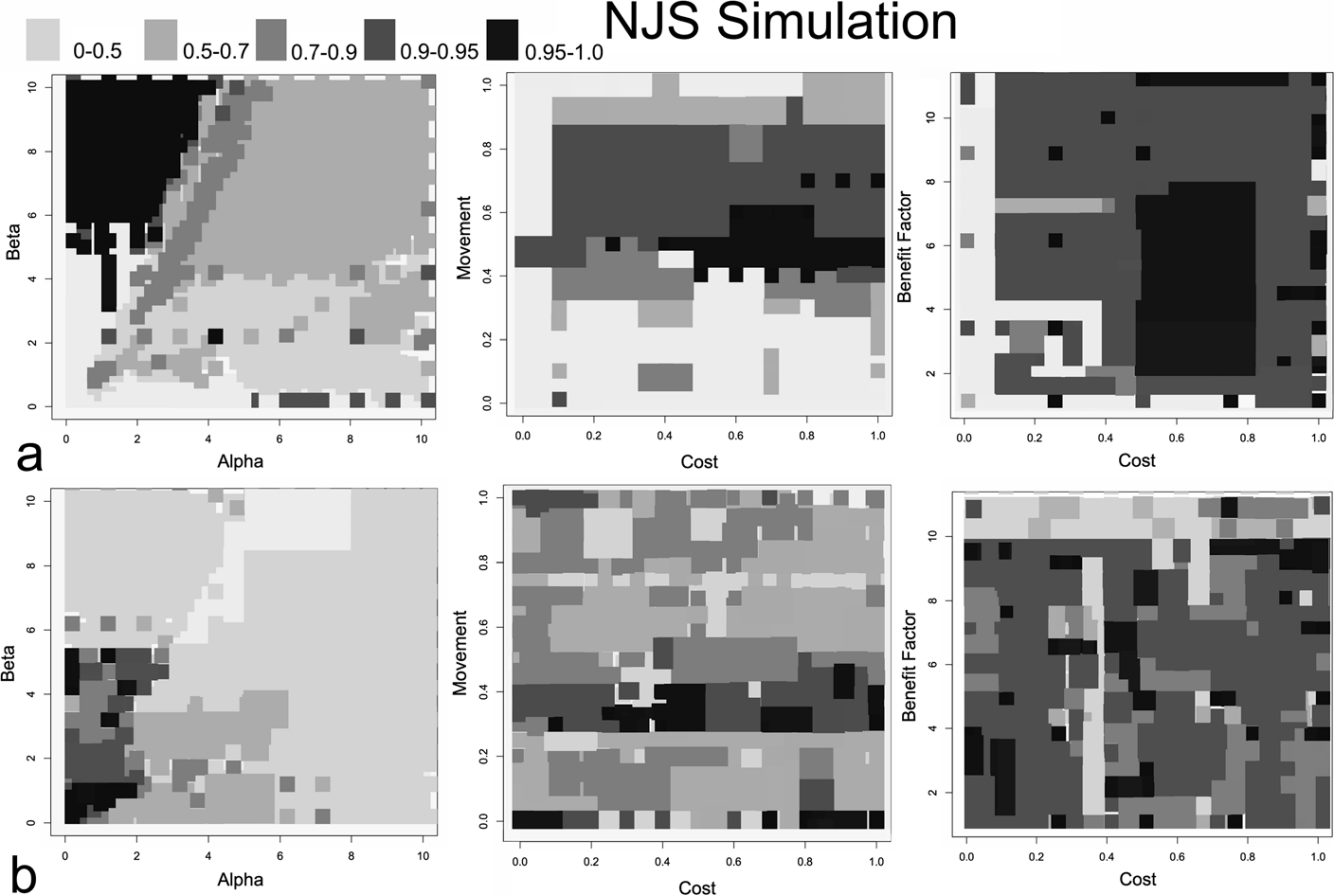
The parameter space for simulated variables for the MBA (**a**) and IA (**b**) in scenario 1. The *top-left ranges and greyscale colors* indicate *r* ^2^ values for parameters

**Fig. 5:**
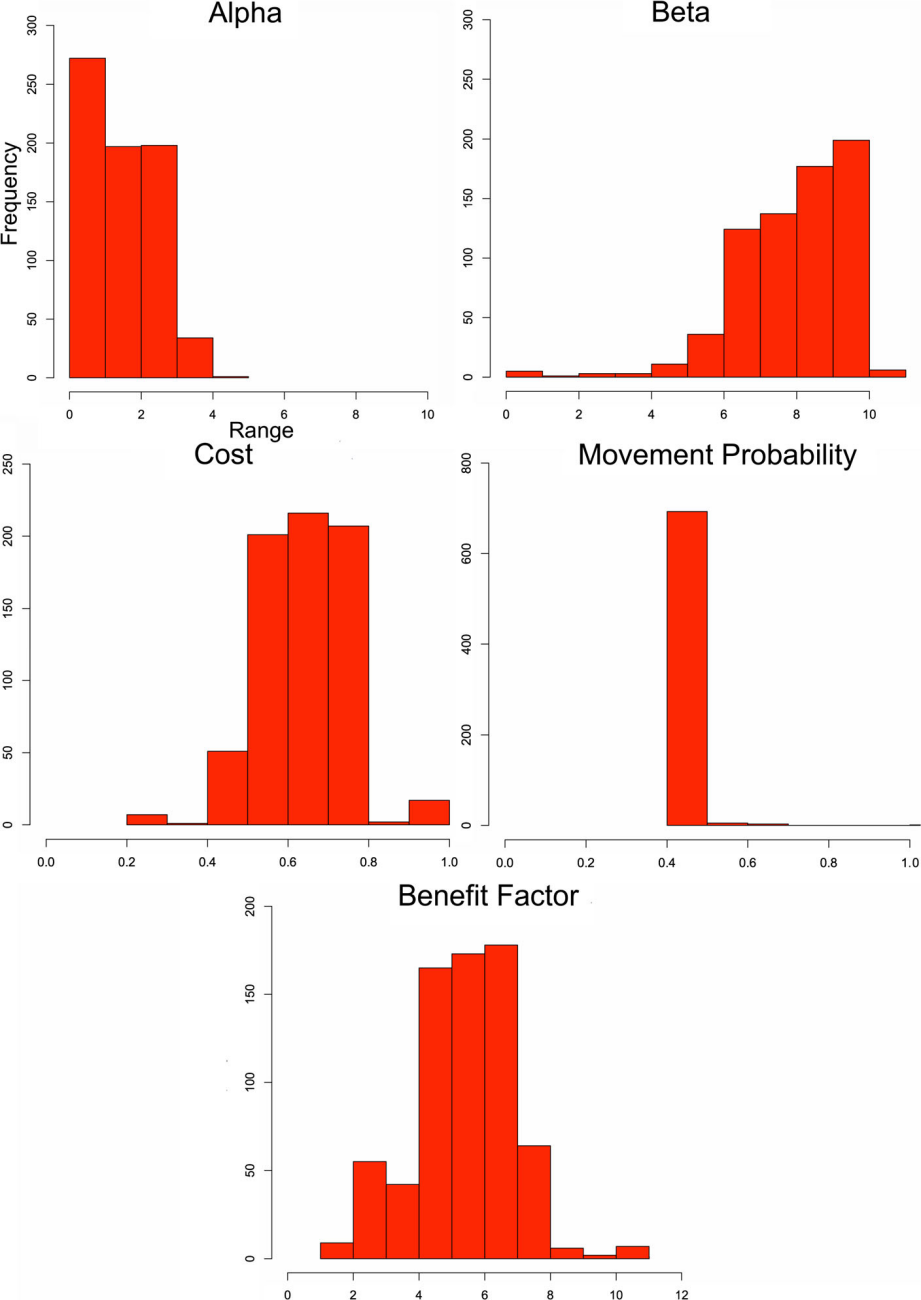
Frequency (*y*-axis) of the five parameters (their names (title) and ranges (*x*-axis)) where *r* ^2^ > 0.98 in a goodness-of-fit test between simulated and empirical MBA NJS sites

**Fig. 6:**
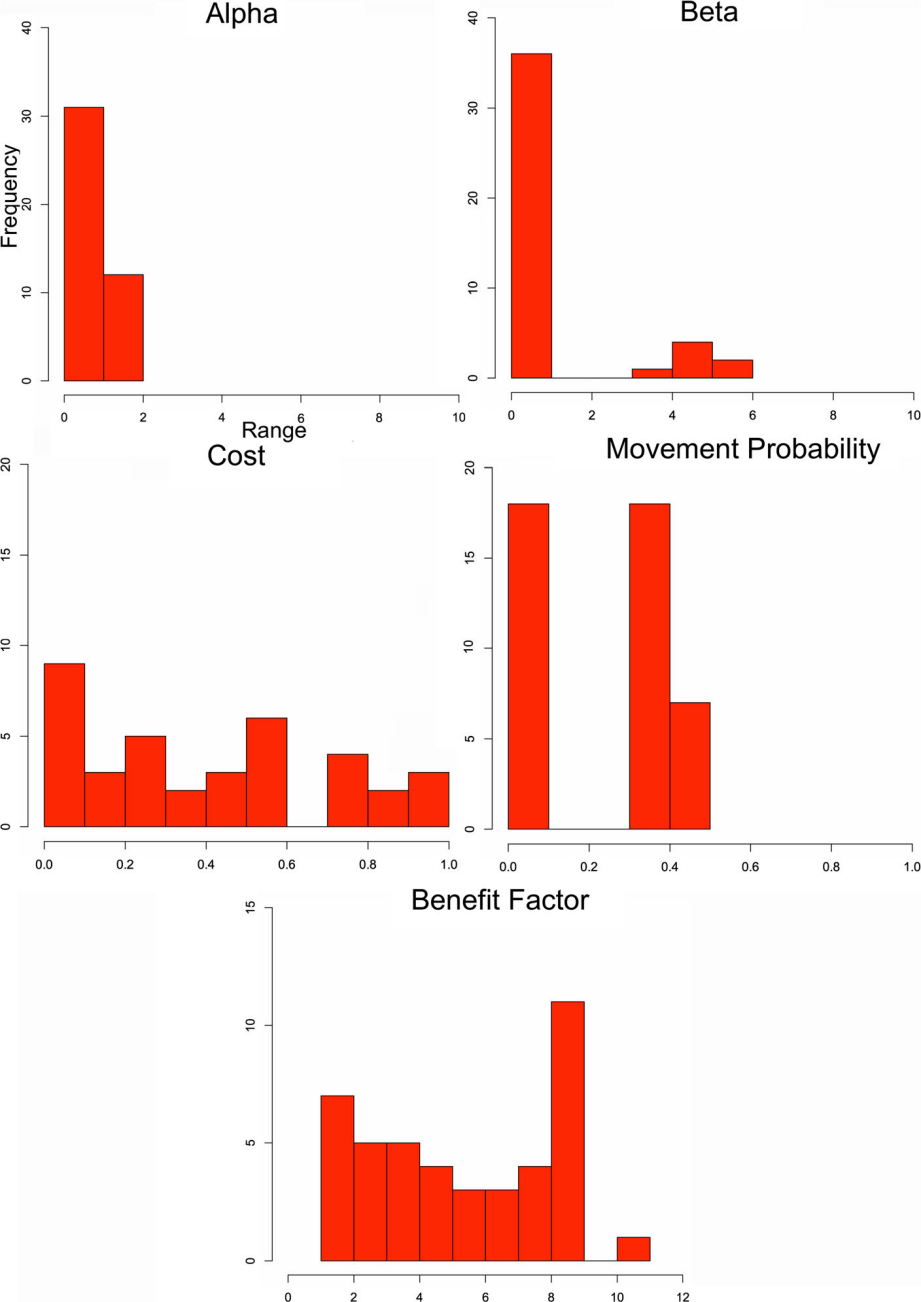
Frequency (*y*-axis) of the five parameters (their names (title) and ranges (*x*-axis)) where *r* ^2^ > 0.98 in a goodness-of-fit test between simulated and empirical IA NJS sites

**Fig. 7:**
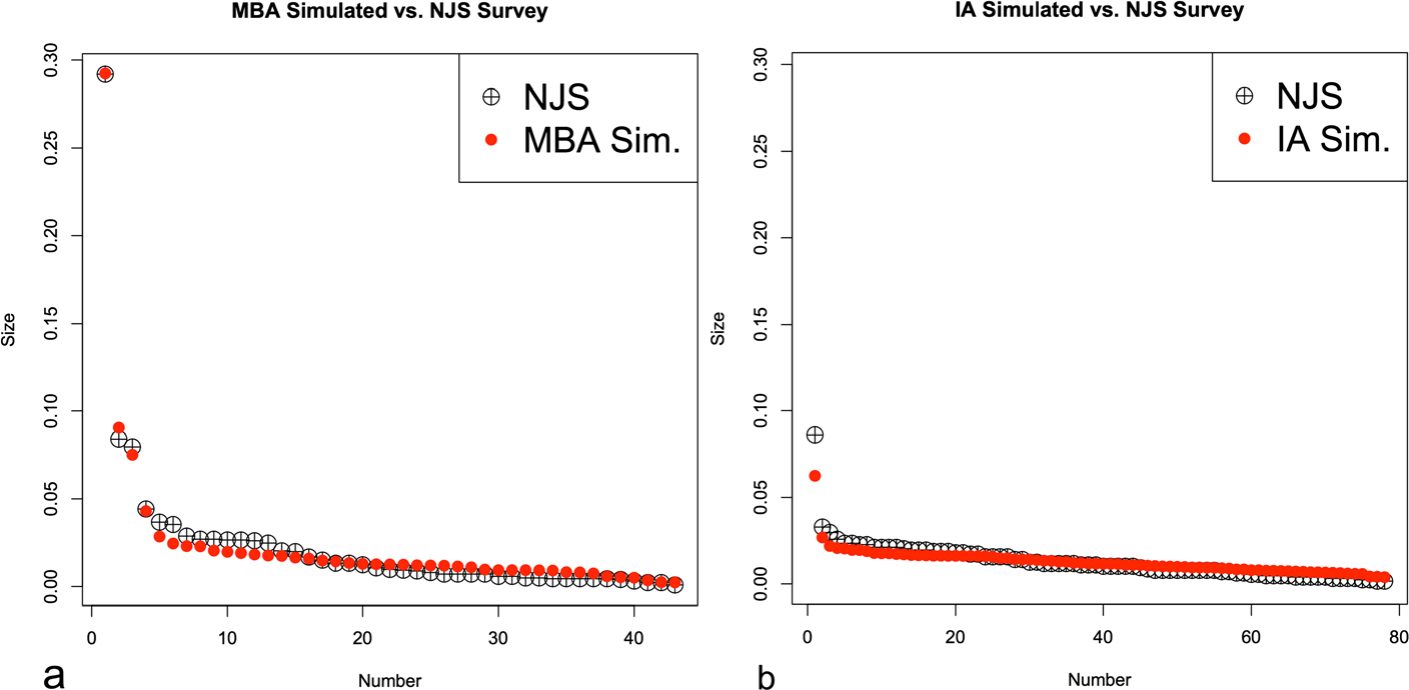
Comparison of population (simulated) to size (empirical) ratios for the MBA (**a**) and IA (**b**) that show closely matching results between simulated and empirical results. The results for (**a**) and (**b**) are *r* ^2^ > 0.99. The MBA parameters here are return on site attractiveness (*α*) = 0.8, ability to move (*β*) = 7.0, cost (*c*) = 0.6, movement probability (*m*) = 0.5, and agent benefits (*b*) = 2.0, while the IA parameters are *α* = 0.2, *β* = 0.7, *c* = 1.0, *m* = 0.36, and *b* = 1.0

Figure 4 shows regression (*r* ^2^) results, ranging from 0 to 1.0 (shaded areas), using an ordinary least squares regression on the ratios of simulated site populations, used as a proxy for simulated size, and surveyed site sizes for the MBA (scenario 1a) and IA (scenario 1b) cases, indicating how well simulated results fit survey results. In essence, Fig. 4 shows which variable settings lead to the simulated population data to match more closely to empirical site sizes, with darker colors indicating greater fit between the simulated and empirical data. Results that show *r* ^2^ > 0.98, that is a relatively high goodness-of-fit between empirical and simulated results for the MBA and IA, are shown in Figs. 5 and 6, respectively. These figures provide the frequency of simulations that have these high fit values, rather than just simply if a setting has a close fit with the survey as shown in Fig. 4. This gives an idea which parameter settings and combinations generally have more close-fitting results. The *r* ^2^ > 0.98 range is found to indicate both a very close visual (i.e., qualitative) and statistical fit, which is why it is used. Each frequency count in Figs. 5 and 6 represents an averaged parameter variation result in which *r* ^2^ > 0.98; there are 702 parameter variations that fall within this threshold in the MBA, while there are only 42 in the IA case. For scenario 1a, the best-fit parameter setting has *r* ^2^ = 0.998, where *α* = 0.2, *β* = 6.6, *c* = 0.8, *m* = 0.5, and *b* = 7. For the IA, the best-fit results (*r* ^2^ = 0.994) are *α* = 1.7, *β* = 4.2, *c* = 0.26, *m* = 0.34, and *b* = 1. Figure 7 shows two examples, one MBA and the other IA, where results had a strong fit with empirical data.

Where there is a close correspondence between simulated and empirical MBA data, Fig. 5 shows that *α* ranges between 0 and 5 closely fit the survey data’s settlement size hierarchy, while the results for *β* are largely between 4 and 10.2. This indicates a relatively moderate to a low emphasis on *α* and greater impediment to movement (i.e., higher *β*) leads to the settlement size hierarchy observed. As for *c*, the range is mostly between 0.4 and 0.8, with some results having a close fit near 0.3 and 1.0. Movement probability (*m*), on the other hand, is almost always near 0.5, showing that a very narrow *m* range allows close-fit results, while well-fitting *b*, or benefits an agent brings to a site, values mostly cluster around 4–8. These results can be interpreted to mean there is relatively moderate to high cost in flow, relative to return on benefits provided by individuals, while very high or low benefits by agents do not often lead to well-fitting results. The benefit factor begins to become relevant when other agents from the same social group, in this case from the same initial site, are found in other sites. Variable *m* shows much greater restriction, around 0.5, and there is a reasonable chance an agent could move if benefits from their current settlement are negative or lower than other sites around them. The movement value is not to the extent where people immediately leave their site, but it shows that movement should occur frequently, even if interactions are mostly across short distances (i.e., moderate to high *β* values).

For the IA, cases that meet the *r* ^2^ > 0.98 values have *α* ranging between 0 and 2 and *β* mostly lower than 1 but also ranging between 3 and 6 to a lesser extent. There is less return on settlement attractiveness than the MBA case, showing less importance on settlement advantages in reinforcing site size, but far less restriction to movement, allowing flow to be more dispersed. For *m*, values range between 0 and 0.1 and 0.3 and 0.5. In essence, very low probability of movement or a moderate probability lead to observed results. This indicates two possible movement range frequencies, rather than just one as in the MBA case, are possible for the IA, where very few people move or more frequent movement is found. This will be further discussed in scenario 2. The other variables appear to be more random or have less of a clear pattern; *c* ranges between 0 and 0.6 and 0.7 and 1.0 seem to lead to the observed simulated results. For *b*, most of the close-fit results range between 0 and 9, with some between 10 and 11. In essence, *α*, *β*, and *m* appear to have narrower ranges in leading to a close fit between simulated and empirical results for the IA case, while the values of the other variables have very wide ranges.

**Fig. 8:**
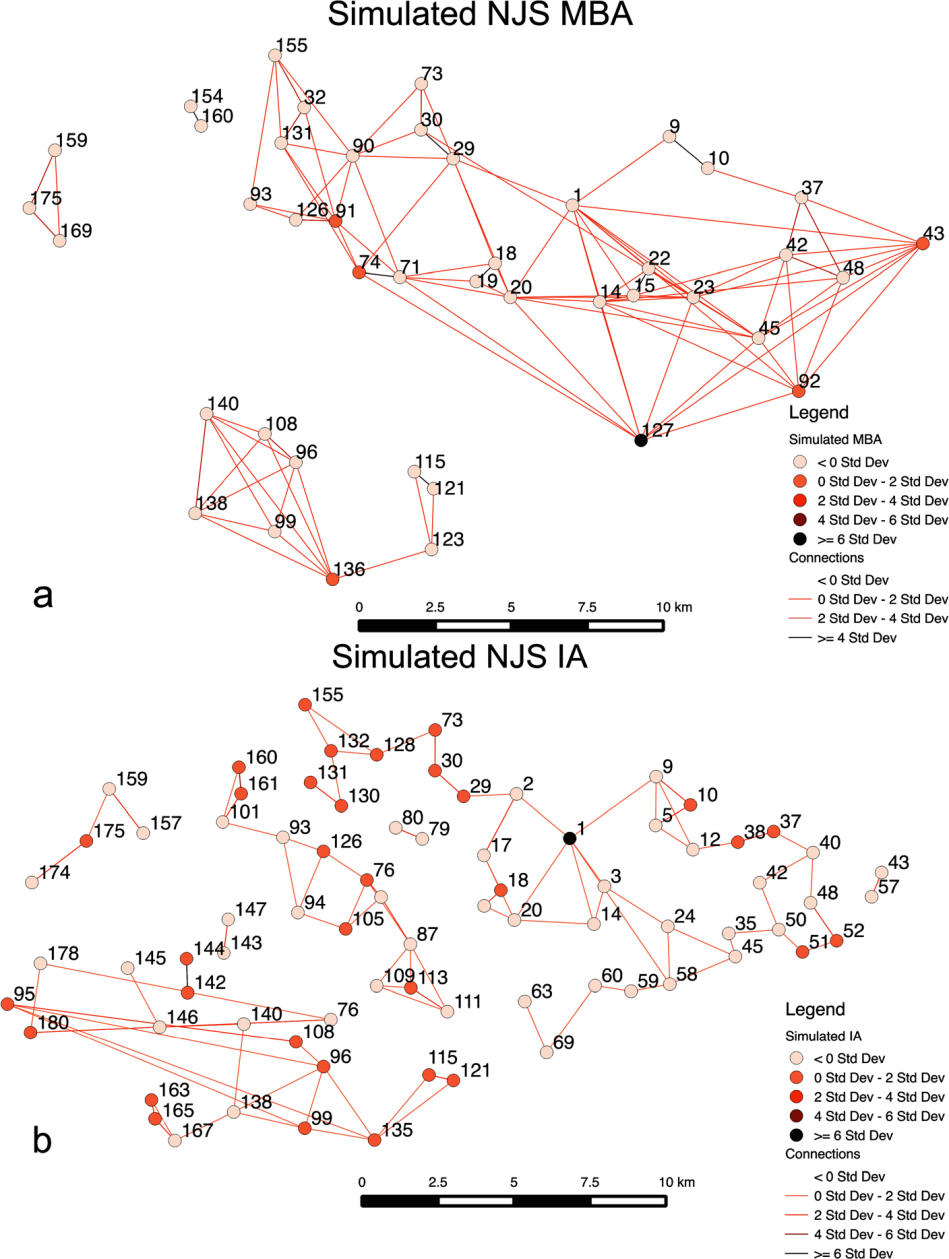
Simulated sites for the MBA (**a**) and IA (**b**) showing site size and predominate interactions, based on the number of times an agent moves from one site to another, derived from a Nystuen and Dacey (1961) network representation. Standard deviation is used to show population variation and number of movements between sites; for movement, where the result is less than 0*σ*, there is no display in order to simplify the visual representation

Figure 8 shows settlement sizes, for two example results that are typical for well-fit results in this scenario, using standard deviation on simulated population to indicate where larger sites are located. The figure also applies Nystuen and Dacey (1961) graphs, as similarly used in Davies et al. (2014), of settlement connections based on movement of people to sties. While Fig. 8a shows site 1, Tell al-Hawa, is not the largest site in simulations, as observed in the MBA of the NJS survey, Fig. 8b does show the IA scenario does sometimes lead to site 1 being the largest simulated site, matching the NJS survey. In Fig. 8a, what is evident is that the MBA case has a large portion of sites with multiple links, showing a high portion of local interactions or movements between neighboring sites, with only two sites not having multiple links, where movement of people is *σ* > 0. In the IA case, the portion of *σ* > 0 links for sites is fewer (63/78 sites). Overall, a greater number of links in the MBA case indicates more overall movement, although much of it is concentrating toward neighboring sites that then connects to larger sites. In the IA, movement is more diffuse and there are fewer hubs attracting a large number of movements. Furthermore, for all in-degree links, the highest number is 24 in the MBA case, while it is 14 in the IA, showing the higher level of local interaction and migration in the MBA case. There are also 12/43 sites with 10 or more in-degree links, while it is 6/72 in the IA.

As for degree centrality, based on total number of movements going to or through a site, site 37 is the most central in the MBA case, while it is site 144 in the IA. If the average and standard deviation for number of movements in links is observed, the results are 742 and 1,213 for the MBA respectively and 877 and 1,682 for the IA, respectively. While this represents the fact there are more people in the IA case, it is evident that there is also more variability in IA movements. On the other hand, the results indicate greater movement of people through different sites in the MBA case (6,730 movements on average versus 4,485 in the IA), as people made their way to the larger sites. For the MBA, people do not simply move to neighboring sites, but movements continue until people reach the larger sites such as site 127, which are more attractive than others, leading to population concentration at attractive sites and greater differences in population between sites. This shows that people did not immediately find the most attractive site, rather the limitations on movement, as represented by *β*, dampened long-distance interactions. In the IA, diffuse movements and lack of attractive sites create more of an even population in the IA scenario, with a higher portion of sites having 0–2*σ* for population. The IA example applies a *m* of 0.36, while Fig. 6 demonstrates it is possible to get well-fit results with a much lower *m* (i.e., less than 0.1). This will be discussed further in scenario 2. Overall, this scenario has demonstrated that the simulation model does create overall site hierarchies that match different periods’ survey results.

### Scenario 2: Size and Rank Matching

Although the first scenario indicates simulations do closely match site size hierarchy between empirically surveyed and simulated sites, matching not only the hierarchy but the ordinal rank in size of observed and simulated sites proves to be more difficult. In other words, the model in scenario 1 shows that model output often does not have a close match between the ordinal rank-size for specific sites. In fact, when the same regression in scenario 1 is applied so that site rank and size are compared with the matching simulated output, the best results are *r* ^2^ values of 0.87 and 0.5 for the MBA and IA, respectively. Such results are of no surprise since geography is what mostly gives sites initial advantages over other sites in scenario 1. This indicates a need to apply additional factors that allow some sites to have initial advantages to enable them to reach greater size than other sites, while allowing a closer correspondence of simulated rank and size for each site. To enable sites to have advantages relative to other sites, *t*, which controls this aspect, is utilized. This variable also has the benefit of accounting for edge effects, as areas outside of the simulation could be providing benefits or disadvantages that sites receive and affecting site interactions.

**Fig. 9:**
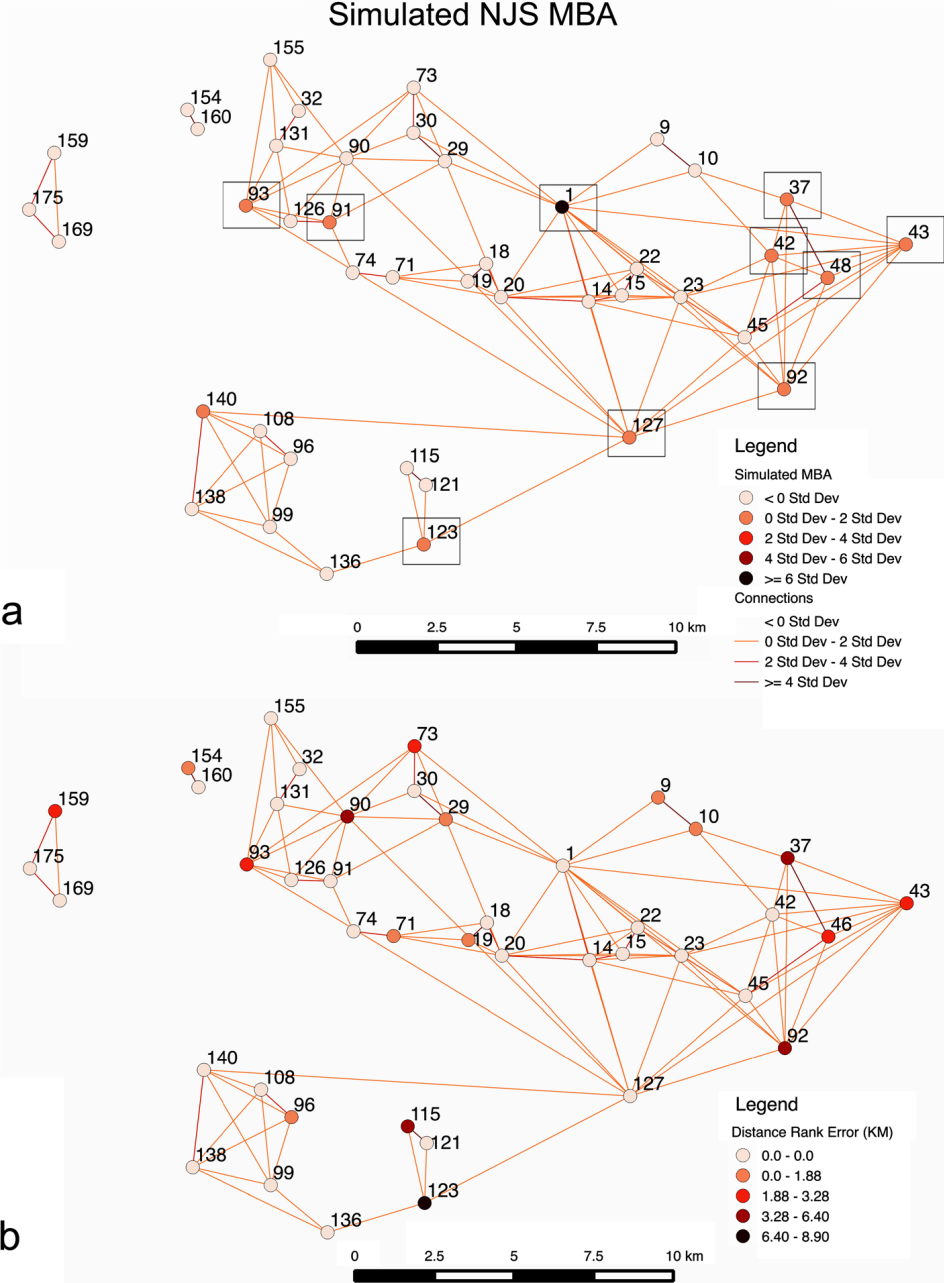
Results indicating *t* value variations (**a**) for the MBA simulation that most closely fits rank and size of the NJS, with the top 10 sites indicated by a *black box*, and distance rank error (**b**) between the simulated and empirical rank categories. *Links* indicate number of movements between sites as expressed using a derived Nystuen and Dacey graph and *σ* values

What is likely evident in the periods studied is that sites did have advantages or benefits that allowed them to become more populated than other sites. A method comparable to Davies et al. (2014) is employed by looking at categories, or ranges, of site’s empirical size estimates in order to create values for *t*. In this case, rather than predetermining the number of categories of size used for *t*, variations of *t* are simulated by testing this parameter to see what the minimal number of *t* value categories, or differences,  are needed so that more than half of the largest ten sites (Table 1) are forecasted by the simulation. The purpose of this approach is that it would demonstrate the model’s capability in forecasting larger sites without overly fitting the model (i.e., many different *t* value categories) and indicates that the model has a far better chance at determining likely larger sites than random chance. For the MBA, four *t* (i.e., endogenous/exogenous benefits given to a site) categories are found to be needed in order to correctly forecast more than half the ten largest sites. In this case, seven of the ten largest sites are forecasted when *t* values are 3, 2, 1, and 0.5 for sites that are >10, 10–5, 5–1, and 1> ha respectively in empirical survey size (Fig. 9a). The result of this in a Spearman’s rank correlation coefficient is 0.61, while a Pearson correlation coefficient test between the simulated and observed site sizes produced 0.94 for the MBA. Both these statistical measures are used because high coefficient values in both tests demonstrate the best rank, which Spearman’s test captures, and size fit, which Pearson’s correlation coefficient indicates. Overall, the results demonstrate that the *t* categories do produce rank-size values that match reasonably the survey record. The best matching parameters (Fig. 9) for the four *t* value categories in the MBA are *α* = 0.4, *β* = 9.7, *c* = 0.4, *m* = 0.5, and *b* = 6.5.

Additionally, looking at the average distance between the observed rank categories, that is the sizes used from the NJS survey to create the *t* values, and simulated rank values, which is what the simulation produces in the rank category of a site, the result is about 1.31 km (Fig. 9b). In this case, this value is called the distance rank error. Therefore, even in cases where the rank of simulated sites did not closely match the observed results, the distance rank error indicates the simulated site is not far from the correct size category. The interaction links for sites, in relation to connectivity of sites, show very similar results and structure to Fig. 8a, with site 14 being the most central based on total number of movements, as the population migrates to the large sites (e.g., site 1). However, the overall distribution of movements per link is nearly identical to Fig. 8a.

**Fig. 10:**
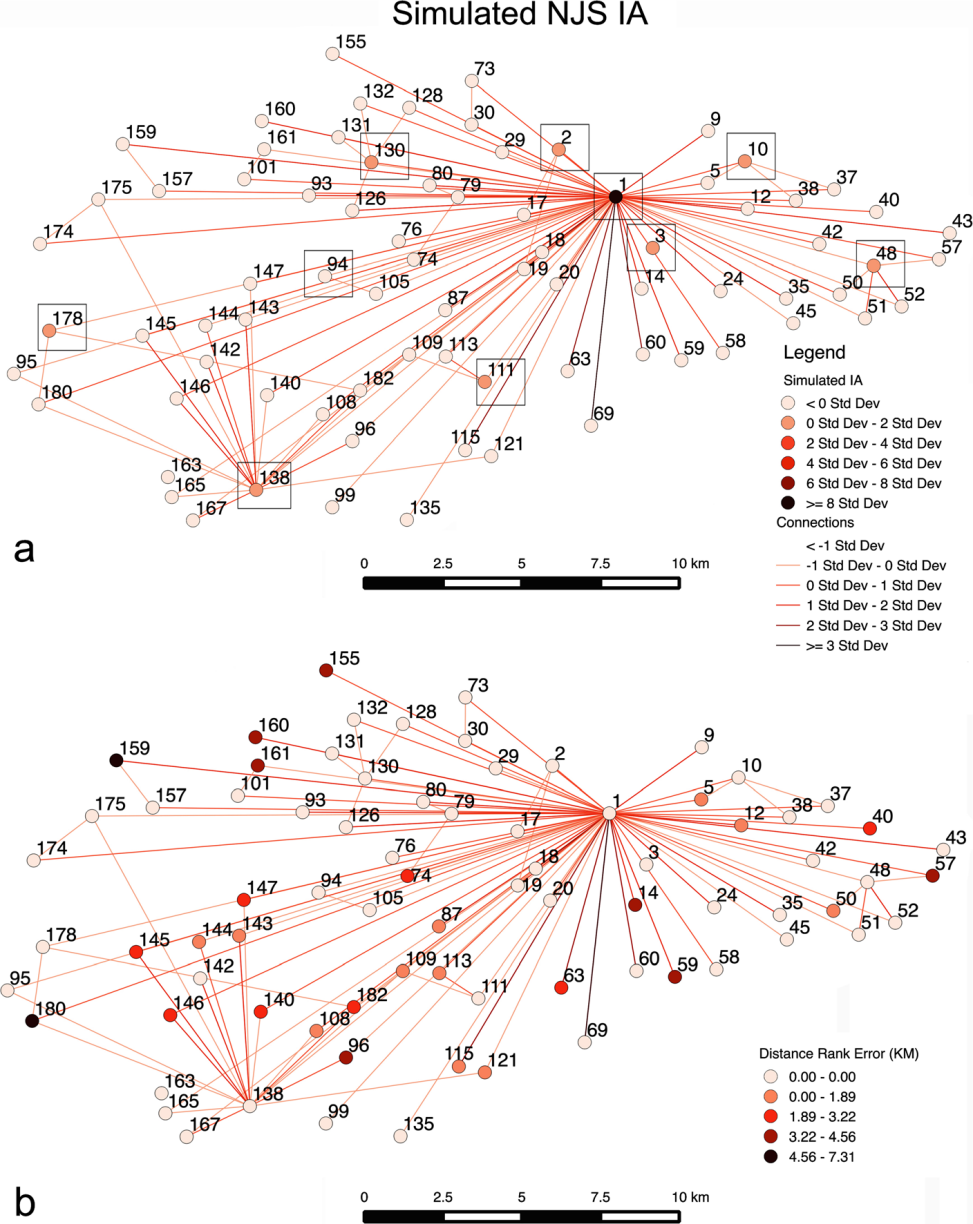
Results indicating *t* value variations (**a**) for the IA simulation that closely fits rank and size of the NJS, with the top 10 sites indicated by a *black box*, and distance rank error (**b**) between the simulated and empirical rank categories. A Nystuen and Dacey graph is used to show movements between sites

For the IA case (Fig. 10a), five categories for *t* are needed to enable a greater than 50 % matching of the ten largest sites. If there are four *t* value categories, 50 % accuracy for forecasting the largest ten sites is achieved, but not greater. Sites ranging in empirical survey sizes of >10, 10–4, 4–2, 2–1, 1> ha with simulated *t* values of 5, 4, 3, 2, and 1, respectively are used here. The best Pearson correlation result, where more than half of the ten largest sites are forecasted, is about 0.84, while the Spearman result is 0.79. This indicates, while the overall correlation is not as good as the MBA case, as densely located sites create more nearby areas where migration maybe drawn to, the Spearman result indicates this case does a better job in reproducing the ranks in the empirical results. In this case, seven of the ten largest sites are forecasted, where *α* = 1.5, *β* = 1.8, *c* = 0.2, *m* = 0.001, and *b* = 2.5. The distance rank error for the IA case is 1.04 km on average (Fig. 10b). Unlike the MBA case and Fig. 8b in scenario 1, the results show a very different interaction link structure, with site 1 having the most interactions by a wide margin, and sites 138 and 48 at a distant second and third respectively in migrations. Site 1 has 77 links, indicating every site interacted with it. What the results suggest is that while *m* is very low, because *β* is relatively low (i.e., it is relatively easy to move) people from throughout the survey area migrate to site 1 directly, rather than through intermediate sites, because of the site’s advantages. Such a structure is similar to what is shown in Fig. 6 (movement probability graph) in scenario 1, which shows that very low *m* probabilities could lead to settlement structures observed for the IA. Mostly, however, *β* values are lower than what is evident in the MBA case. In essence, Fig. 10b highlights that a second model, one where there is low *m*, can lead to structures observed in the IA, in addition to what is shown in Fig. 8b. This case indicates that when movement does happen it is focused on a site with advantages with distance not being a major factor.

### Scenario 3: Survey Sampling and Robustness

At any given time, only a subset of the surveyed sites may have existed within the periods studied, as survey results may not be able to clearly identify subperiods within the MBA and IA. To ameliorate a situation where sites may have not been contemporary, and to assess the robustness of the results achieved earlier through random sampling, a repeated sampling approach is applied where only a portion of sites is executed in a given simulation run (i.e., a bootstrapping method). This portion of sites is sampled using a range of probabilities, where a given site will not be in a simulation run, that are 1/5, 1/3, 2/5, and 1/2, with each of these variations run for 500 different simulation runs for the MBA and IA cases using the parameter settings from the results in scenario 2. The results are then averaged for all sites so that an overall rank-size hierarchy is achieved, even though not all sites are simulated and the combination of sites differs in each simulation run. This approach allows us to see how sensitive results are when sites are removed from simulations and to see if the overall patterns observed in the last scenario are relatively meaningful and reproducible by seeing if similar patterns are achieved in this scenario.

**Fig. 11:**
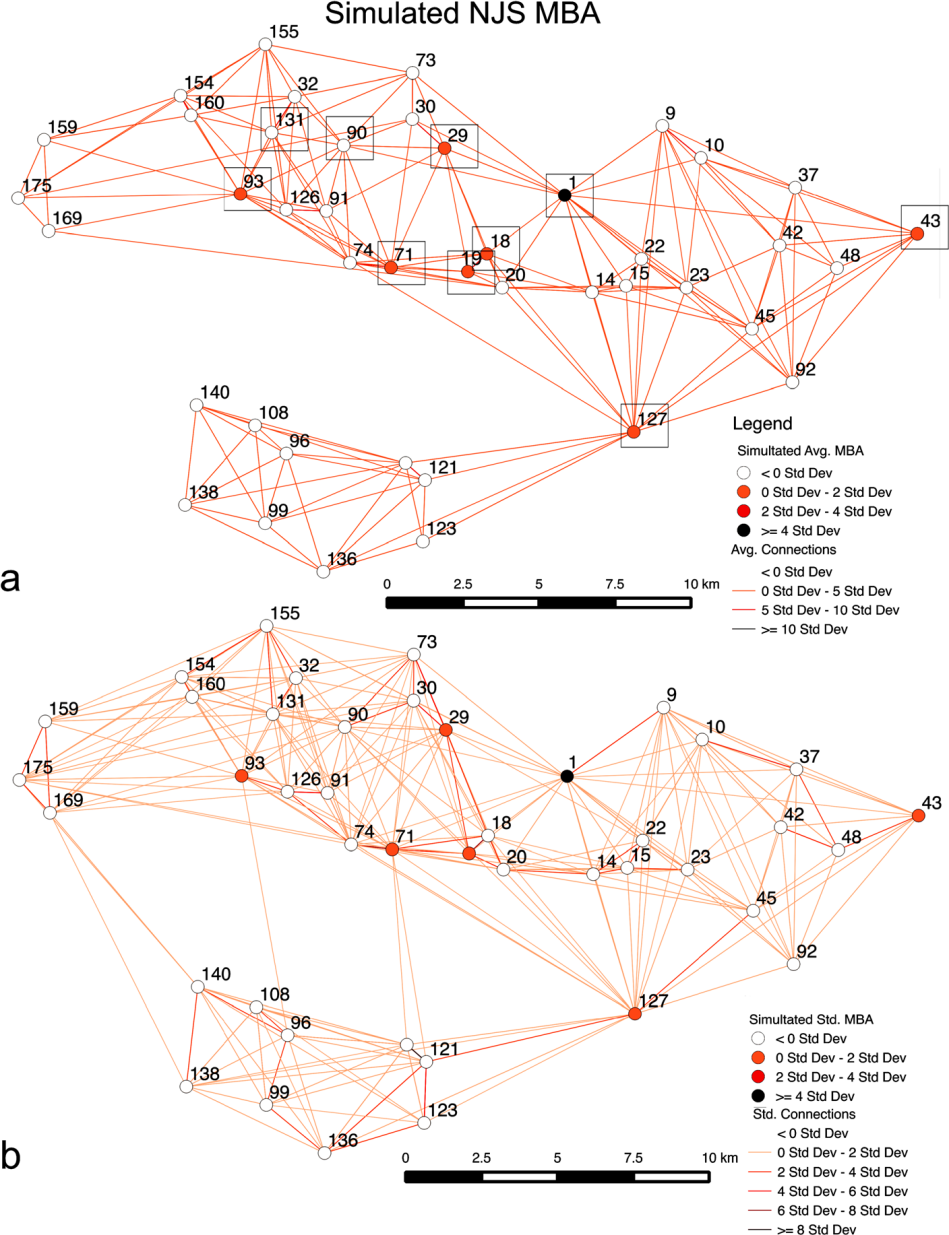
MBA case where each site has a 1/3 probability of not being in a simulation run. The simulated average populations and migrations between sites for 500 runs are indicated (**a**) along with the top 10 simulated sites. The *σ* for different simulation runs for population and migration between sites is given as well (**b**). Similar to earlier cases, a Nystuen and Dacey graph is used along with *σ* values

The results for these probability scenarios, for both cases, are given in Table 3; as before, both Spearman’s and Pearson’s correlation coefficients are given, as this provides stronger rank and size correlations. For the MBA, the 1/5 and 1/3 probabilities show a relatively strong Pearson’s *r* value, while the 1/2 probability indicates a large decrease in this value. Nevertheless, the Spearman’s correlation coefficient value is relatively consistent, indicating that the rank order stays relatively stable between scenarios. In all cases, more than five of the ten largest sites are forecasted; in fact, the weakest Pearson’s r correlation did very well in forecasting the largest sites, even if the site size hierarchy results are weaker than other cases. Overall, the results show that the rank and size hierarchy of sites is maintained fairly well and relatively comparable to the empirical data until the simulation has more than 40 % of the sites missing at any given time. The Spearman’s rank correlation coefficient and number of top 10 sites forecasted gives some confidence that the results achieved in scenario 2 are meaningful even if part of the dataset is used. Figure 11 indicates MBA output, which is the 1/3 probability case, which has the best correlation coefficients for scenario 3. Results here show that sites 1, 43, 93, and 127 are forecasted to be in the top 10 largest in both scenarios 2 and 3. One possible interpretation is that the results suggest most of these sites would have been long-lived and contemporary, as the overall rank and size hierarchy are more closely maintained if many or all sites are present in a given scenario. Results in Fig. 11 indicate interactions that are somewhat similar to what is observed in Fig. 9; however, the main difference is there are more varied links with greater than 0σ movements, which represents the variability of movements from case to case due to some sites being removed or added based on the probability. For the overall average, the most central node is site 30, followed closely by sites 19 and 18, respectively. While these results are different from what is seen in scenario 2, structurally they are similar as sites near site 1 play an important conduit role in moving people closer to the high population sites. Movements are also seen to be mostly between nearby sites, with movements averaging 4.75 km distance.

**Table 3:** Results from scenario 3 testing for sampling and robustness of the modeled survey region’s cases

Probabilities	0	1/5	1/3	2/5	1/2
MBA Pearson's *r*	0.94	0.9	0.93	0.82	0.46
MBA Spearman's *ρ*	0.61	0.53	0.54	0.45	0.48
MBA number of sites in top 10	7	6	7	8	9
IA Pearson's *r*	0.84	0.82	0.85	0.86	0.88
IA Spearman's *ρ*	0.79	0.73	0.75	0.83	0.79
IA number of sites in top 10	7	4	6	5	6

The results from scenario 2 are also indicated under the “0” column

**Fig. 12:**
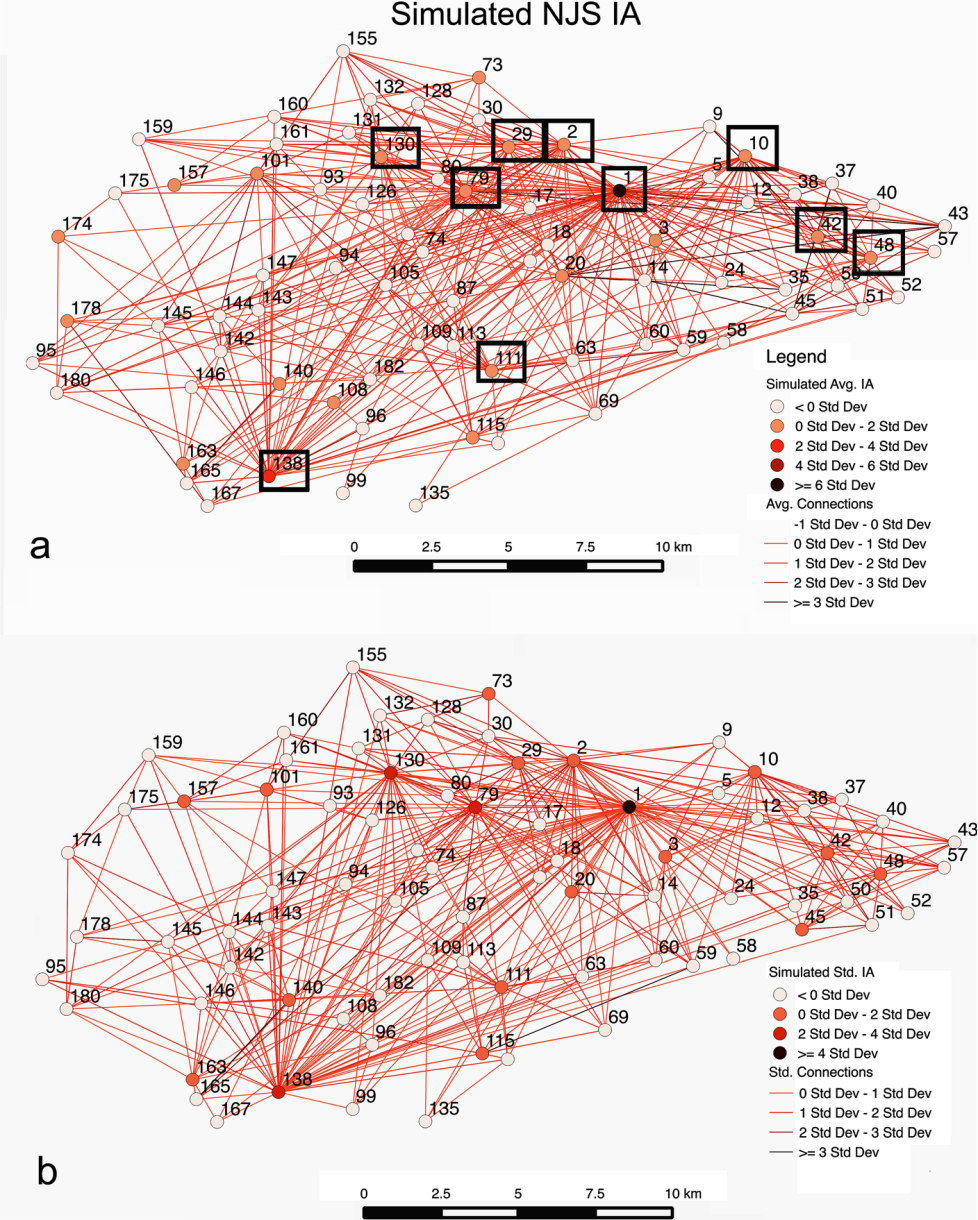
IA case using a Nystuen and Dacey graph where each site has a 1/2 probability of not being in a simulation run. The simulated average site populations and migrations for 500 runs are indicated (**a**) along with the top 10 simulated sites. The *σ* values for population and migration between sites in the simulation runs is given (**b**)

For the IA (Table 3), the Pearson’s *r* value is 0.88 when 1/2 of the sites are not simulated in a given run, with improving Pearson’s *r* values greater than 1/5 probability for sites not being in simulation runs. In addition, the Spearman’s rank correlation coefficient value improves for probability values between 1/5 and 2/5 of sites not simulated in runs. However, in the two cases, it is evident that forecasting the top 10 sites is not always greater than five. Figure 12, which has 1/2 probability, indicates the scenario with the best correlation coefficients and most forecasted top 10 sites. This output is a reflection of the greater variability found between runs in the scenario from case to case. Despite the fact that Fig. 12 appears to show more noisy interactions, for both scenarios 2 and 3 in the IA, sites 1, 2, 10, 48, 111, 130, and 138 are forecasted to be among the largest ten sites. This case shows many interactions where movement is greater than 0*σ* for links, which is once again a reflection of the variability found in given runs. However, looking at the overall average, and very similar to what was seen in Fig. 10 in scenario 2, site 1 is the most central as people are able to travel relatively farther distances to an attractive site. Site 138 is the second most central, as it is in scenario 2, where it forms a smaller regional center to the southwest of site 1. As with scenario 2’s IA case, many movements are long-distance and not just between sites next to or very close to each other. Interactions, or movements of people between sites, are on average covering 9.36 km in the IA in Fig. 11, indicating much more distant interactions than the MBA case. Although the IA case seems to forecast fewer of the top 10 largest sites, Pearson’s *r* and Spearman’s *ρ* values suggest there is a good degree of confidence in the results achieved in scenario 2. In fact, the results could suggest that many of these sites were not contemporary and existed for shorter periods within the IA, as the Pearson’s *r* value improves in cases where the probability of a site not being in a simulation increases, while Spearman's *ρ* is best when 2/5 of the sites are removed. Admittedly this is speculative; however, the results do suggest that the rank-size hierarchy demonstrated in scenario 2 appears to be a meaningful pattern as comparable or even better results are achieved via subsampling.

## Discussion

This presentation has given a number of results that highlight the main goal of this research, which is demonstrating how a model could integrate site-specific factors and agent choice that enable rank-size hierarchies to be achieved that are comparable to the empirical record. At a general level, scenario 1 investigates parameters used in modeling, indicating that some parameters require specific ranges in order to closely replicate settlement size patterns for the MBA and IA. Scenario 2 demonstrates the model’s ability to forecast the correct largest sites and maintain relatively close fit with rank-size values without overly fitting endogenous/exogenous site benefits (*t*) values to scenario runs. Scenario 3 demonstrates that for probabilities less than 40 %, where sites are removed from simulation runs, rank-size hierarchies from scenario 2 are fairly robust and maintained, for the MBA case, suggesting scenario 2’s results are likely to be meaningful. In the IA case, as the probability increased toward 40 or 50 %, the correlation coefficient results appear to improve. While these results could suggest that most or almost all sites in the MBA were contemporary, the IA case may suggest that a good number of the sites were short-lived and not contemporary.

Specific archaeological benefits from scenarios are evident from the results. The *α* setting in the MBA and IA cases, specifically in scenario 1, shows that in general there appears to a greater emphasis on feedbacks to site attractiveness in the MBA, leading to some sites, that is those sites with more benefits (i.e., flow) than others, to become even larger than other sites, leading to greater differentiation and hierarchical differences in site size. Scenario 2 shows that site advantages, such as *t*, do not need to be so great for major sites (e.g., site 1 Tell al-Hawa) to gain major population advantages over their neighbors, while the IA case in scenario 2 shows that relatively greater *α*, at least compared with results from scenario 1, could be needed for site 1 to gain a great enough advantage to enable differentiation from its neighbors. This might be because there are more sites in the IA scenarios, which leads to more settlements attracting flow and movement away from other centers. Scenario 3 for the IA does suggest a possibility that many sites may not have been contemporary. A relevant result in scenario 2 is that in both the MBA and IA cases, the model only needed a few size categories to forecast a large portion of the top 10 largest sites and achieve results with relatively good fit to their empirical rank-size hierarchy. This result indicates some role in geography, as sites that are well positioned between sites could benefit more greatly with nearby interactions; however, geography is not necessarily a dominant factor, at least at the survey scale, in leading to specific sites becoming relatively large (e.g., site 1 in the MBA and IA), as *t* is used for consistent site advantages and to forecast most of the largest sites.

For the MBA and IA, *β* is shown to play an important role in limiting or facilitating movement across the modeled region. For the MBA, as shown in scenario 1, high *β* values indicate more local or neighboring settlement interactions, while for the IA lower *β* values enable diffuse or easier and more distant interactions to occur. This situation could be reflected by the hollow-ways, or remnants of ancient roads, found in the survey area and larger region, where many short-distance routes appear to develop by the Early Bronze Age (Wilkinson 1994; Ur 2003), indicating numerous local interactions. While long-distance hollow-ways are found in the Bronze Age, by the IA long-distance roadways appear to become more significant (Altaweel 2008), which may reflect a period where long-distance movement was easier and thus flow or migration of people could have become more dispersed in regions. This could be driven by the political situation in the two periods. In the Bronze Age, and particularly evident in the MBA, nearby states and communities were often or were likely to be in conflict or lacked political cohesion (Dalley 2002; Hamblin 2006; Eidem 2012). In the IA, much of this period is dominated by a singular empire, in the form of the Neo-Assyrians that controlled vast regions from their capitals in the Assyrian heartland (Radner 2011), allowing for relatively greater political stability and socio-political cohesion within the NJS and greater ease of movement across northern Mesopotamia. In addition, this could explain why *c*, or the cost value, is often high in order to develop settlement size hierarchies similar to the MBA, while in the IA *c* mattered less and could be at different settings.

Individuals, or in this case households, play an important role in shaping site size hierarchies, where choice of movement is affected by common social connectivity and perceived benefits elsewhere (method #5). Agents bring benefits (*b*), but their movement ability (*m*) is also possibly limited regardless of choice to move. Simulated benefits by individuals include economic or social benefits brought by migrations to settlements, where texts indicate how common households are found to play vital economic and social roles in defining urban structures throughout Mesopotamia (Van der Mieroop 1999), while movement, even if desired, is not always possible and the rate and choice of this factor is found to be important in all scenarios modeled. Agent benefits (*b*) appears to cluster between 4 and 8 in the MBA, but the pattern is less clear in the IA and a wider range is found. In scenarios 2 and 3, *b* helps enable sites to achieve positive feedback growth, where the relatively high *b* values enables site 1 and other MBA sites with initial advantages to grow more rapidly.

In the MBA case, a very narrow range near 0.5 for movement probability (*m*) is found to be most relevant, while in the IA case at least two ranges (less than 0.1 and between 0.3-0.5) seem to create patterns noticed in the empirical record. The MBA case reflects that although movements are generally restricted to local interactions, it was relatively easy to move in these shorter distances *m*. For the IA, there are two clear movement patterns. One is probability ranges comparable to the MBA scenario, which creates in this case small settlements of relatively even sizes as the population moves more evenly across the landscape, while an alternative case, as emphasized by scenarios 2 and 3, is a very low *m*. This creates a situation where overall movement is low; however, because *β* is low, when movement occurs, it often happens over longer distances. This leads to a site with clear advantages (i.e., site 1 in the IA NJS) getting a relatively high number of people migrating to it.

Summarizing the variable value ranges and their qualitative interpretation that could be suggested by the results, for the MBA case, *α* suggests greater return to settlement attractiveness, particularly larger sites, while *β* indicates greater restrictions to movement, although people could move somewhat frequently over relatively short distances, as shown by *m.  Agent benefits (b*) and high *c* enabled sites to begin to differentiate their size and rank relative to other sites. All these variables and values could reflect that the numerous conflicts or lack of socio-political cohesion in the MBA for the region may have had an effect by constraining movements of people and goods to shorter distances, while encouraging a greater portion of them to live in larger sites. The IA case shows it is possible for empires, such as the Neo-Assyrian state, to facilitate movement and spread populations, making settlements more equal in size and deemphasizing settlement in larger sites through unifying regional authority, social integration, or pacifying a given area, with mostly low *α* and *β* demonstrating this. For the IA, the other variables all indicate that they either mattered less or reflect easier movement, even if it was infrequent ; *c*, for instance, had wide value ranges, while *m* had more specified ranges, whereby the rate of movement based on probability is either low or somewhat moderate. The case study results are comparable to what is observed in Davies et al. (2014), where conflict socio-political cohesion, with their presence or absence, are suggested to be major driving forces in shaping settlement structures in northern Mesopotamia, with the historical data supporting these possibilities (Dalley 2002; Radner 2011; Eidem 2012).

Factors dealing with settlement and individual advantages, agent choice, cost of transport, facility, or ease of movement have all been considered to be critical in the shaping of Mesopotamian cities and urban growth (Adams 2001; Algaze 2008), which are demonstrated in MBA and IA cases. Other factors, such as *c* in the IA, at times seem to have less of a clear impact or have a wider range of possibility. Overall, the model demonstrates how agent-specific factors, decisions, settlement influences, geography, and outside influences could shape site size hierarchy. As discussed, households and major institutions likely played an important role in shaping urbanism in northern Mesopotamia (Wilkinson et al. 2007; Ur 2010). This model demonstrates how these entities could be studied for their influence on settlements.

## Conclusions

There are several broad benefits demonstrated by the case study and model presented. The model points to a theoretical merging of top-down and bottom-up factors that can be studied together to inform about archaeological problems relating to regional populations and settlement. Many of the factors introduced by the model have been discussed as critical for the development of modern urban systems (Batty 2008), as economic, geographic, and transport factors are utilized in a general way and also play key roles in past urban systems. Theoretical complexity in the past likely plays a significant role in how urban forms develop in Mesopotamia and beyond (Adams 2001), while this perspective is having an increased role in archaeology in general (Bentley and Maschner 2008). Adaptations by learning or simple choice by agents as systems evolve help to shape how the overall settlement system develops. The model presented here merges bottom-up and top-down factors because strengths are found in each methodology, where system-level equations and agent choice generalize and capture the larger behaviors of the system but also inform on how individual choice could shape dynamics and settlement hierarchies as demonstrated here. Further benefits, as already demonstrated by Bevan and Wilson (2013), include models such as this and other entropy maximization types that could be created to begin to forecast regions where larger and smaller sites could have developed. Overall, the modeling approach provides a way to explain why differential growth is seen or expected in the empirical record.

Shortcomings are found in this article, which suggest possible future research areas. This includes not differentiating human agents in each simulation run. While stochastic choices are used to represent varied factors and choices made by agents, with settlements acting as entities or even pseudo-agents that agents can interact with based on their advantages and disadvantages, in reality there would have been different types of individuals and households concurrently operating that could be represented by types of agents through differences in agent advantages (*b*). One of the strengths of an ABM is its ability to represent varied agent types together. In this paper, agents are averaged into a single type per run and multiple types are run in different simulations, as this helps to address uncertainty about the ranges of types that could be found while also controlling for effects by different agent types. Nevertheless, multiple agents can be represented in the same simulation to see how these types could interact and affect settlement hierarchies. Further research in this area seems to be a likely way forward. Additionally, more work can be done to better define how social relationships could affect results. While an easy way to represent potentially complex social relationships is to assign people from the same settlement to be more socially similar, other methods could prove to be more effective in representing or replicating complex social relationships.

Overall, this presentation has brought forth several case-specific and broad benefits that can benefit archaeology in northern Mesopotamia and beyond. With increased use of complexity theory to explain how urban systems form, the paper presents a formal method that can be used to explain this theory in a manner that closely replicates the settlement record. Finally, as discussed previously (Falconer and Savage 1995), the importance of rank-size hierarchies and their use for analysis are dependent on sampling procedures that capture sites at different parts of site size scales, which emphasizes the importance of detailed and intensive surveys such as that found in the NJS. With further surveys applying comparable approaches to fieldwork, these cases should in the long run improve modeling methodology applied to settlement size hierarchies.

## Electronic Supplementary Material

Supplementary Data: https://iris.ucl.ac.uk/iris/publication/962169/1
